# Predicting protein-ligand interactions based on bow-pharmacological space and Bayesian additive regression trees

**DOI:** 10.1038/s41598-019-43125-6

**Published:** 2019-05-22

**Authors:** Li Li, Ching Chiek Koh, Daniel Reker, J. B. Brown, Haishuai Wang, Nicholas Keone Lee, Hien-haw Liow, Hao Dai, Huai-Meng Fan, Luonan Chen, Dong-Qing Wei

**Affiliations:** 1 College of Life Science and Biotechnology, Shanghai Jiao Tong University, 800 Dongchuan Road, Shanghai, 200240 China; 2 Cellular Networks and Systems Biology, University of Cologne, CECAD, Joseph-Stelzmann-Strasse 26, Cologne, 50931 Germany; 3 Department of Genetics, Harvard Medical School, Boston, MA 02115 USA; 4 Wellcome Sanger Institute, Wellcome Genome Campus, Hinxton, Cambridge, CB10 1SA UK; 5 Department of Medical Genetics, School of Clinical Medicine, University of Cambridge, Cambridge, CB2 0QQ USA; 6 David H. Koch Institute for Integrative Cancer Research, Massachusetts Institute of Technology, Cambridge, MA 02139 USA; 7 Division of Gastroenterology, Department of Medicine, Brigham and Women’s Hospital, Harvard Medical School, Boston, MA 02115 USA; 8 MIT-IBM Watson AI Lab, Massachusetts Institute of Technology, Cambridge, MA 02139 USA; 9 Laboratory of Molecular Biosciences, Life Science Informatics Research Unit, Kyoto University Graduate School of Medicine, Kyoto, 606-8501 Japan; 10 Department of Computer Science and Engineering, Fairfield University, Fairfield, Connecticut 06824 USA; 11 Department of Biomedical Informatics, Harvard Medical School, Boston, MA 02115 USA; 12 The Gurdon Institute, University of Cambridge, Tennis Court Road, Cambridge, CB2 1QN UK; 13 Center for Genome Sciences and Systems Biology, Washington University, St. Louis, MO 63130 USA; 14 Key Laboratory of Systems Biology, Innovation Center for Cell Signaling Network, Institute of Biochemistry and Cell Biology, Shanghai Institutes for Biological Sciences, Chinese Academy of Sciences, Shanghai, 200031 China; 15 School of Life Science and Technology, ShanghaiTech University, Shanghai, 201210 China

**Keywords:** Computational models, Computational models, High-throughput screening, High-throughput screening, Drug screening

## Abstract

Identifying potential protein-ligand interactions is central to the field of drug discovery as it facilitates the identification of potential novel drug leads, contributes to advancement from hits to leads, predicts potential off-target explanations for side effects of approved drugs or candidates, as well as de-orphans phenotypic hits. For the rapid identification of protein-ligand interactions, we here present a novel chemogenomics algorithm for the prediction of protein-ligand interactions using a new machine learning approach and novel class of descriptor. The algorithm applies Bayesian Additive Regression Trees (BART) on a newly proposed proteochemical space, termed the bow-pharmacological space. The space spans three distinctive sub-spaces that cover the protein space, the ligand space, and the interaction space. Thereby, the model extends the scope of classical target prediction or chemogenomic modelling that relies on one or two of these subspaces. Our model demonstrated excellent prediction power, reaching accuracies of up to 94.5–98.4% when evaluated on four human target datasets constituting enzymes, nuclear receptors, ion channels, and G-protein-coupled receptors . BART provided a reliable probabilistic description of the likelihood of interaction between proteins and ligands, which can be used in the prioritization of assays to be performed in both discovery and vigilance phases of small molecule development.

## Introduction

Exploring protein-ligand interactions is essential to drug discovery and chemical biology in navigating the space of small molecules and their perturbations on biological networks. Such interactions are essential to developing novel drug leads, predicting side-effects of approved drugs and candidates, and de-orphaning phenotypic hits. Therefore, the accurate and extensive validation of protein-ligand interactions is central to drug development and disease treatment. Experimentally determining and analysing protein-ligand interactions can be challenging^1,2^, often involving complex pull-down experiments and orthogonal validation assays. Therefore, multiple efforts have been dedicated to developing rapid computational strategies to predict protein-ligand interactions for prioritizing experiments and streamlining the experimental deconvolution of the interaction space. For example, docking simulations, in which the 3D-structure of the target is used to evaluate how well individual candidate ligands bind to a structure, have been productively applied to identify novel interactions between clinically relevant targets and small molecules^3,4^. Appreciably, docking simulations are unfeasible when 3D structures of targets (e.g., those derived from crystallization and X-ray diffraction experiments) are not available, as exemplified by many G protein-coupled receptors (GPCRs), which are membrane-spanning proteins that are inherently difficult to crystallize. Conversely, ligand-based methods (e.g., fingerprint similarity searching, pharmacophore models, and machine learning approaches) are increasingly applied in research and development for the prediction of on- and off-target interactions, but often require large amounts of available ligand data to achieve the desired predictive accuracy. Another widely used computational strategy is text mining, which uses databases of scientific literature such as PubMed^5^. Text mining relies on keyword searching and is limited in its capability to detect novel bindings. The process can be further complicated by the redundancy of compound or protein names in the literature^6^.

Recently, to circumvent the shortcomings of the ligand- and target-based methods and to benefit from all available information, computational chemogenomics (or proteochemometric modelling) has emerged as an active field of predictive modelling. Here, the study of protein-ligand interactions simultaneously combines the protein target and ligand information with machine learning approaches to provide valuable insights into the interaction space. For example, several methods exist that are capable of predicting target protein families and binding sites based on the known structures of a set of ligands^7–10^. However, with scant information about the actual proteins, predicted interactions are, at best, only between the known ligands and different protein families. Some approaches, which are target-centric, make full use of the protein features, but fail to predict interactions of orphan ligands as the latter have no known links to any proteins^11^. Several methods have been proposed to consider both the protein sequences and ligand chemical structures simultaneously in prediction^12,13,42^.

**Figure 1:**
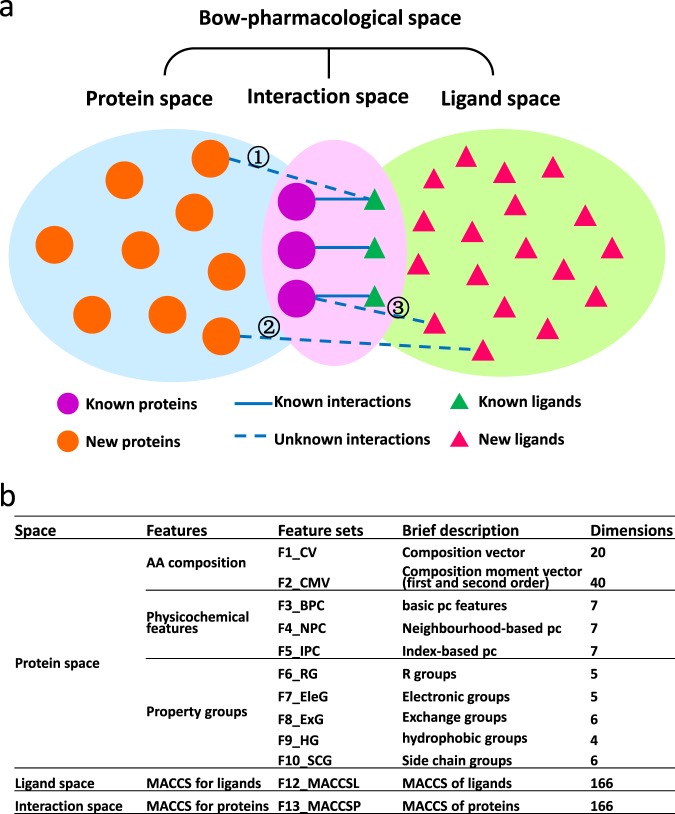
Bow-pharmacological space. (**a**) The bow-pharmacological space spans three subspaces: protein space in blue, ligand space in green, and interaction space in pink. Filled circles represent proteins and triangles represent ligands. Protein–ligand pairs of known interactions from published databases are denoted as “known” whereas those not curated in the databases are denoted as “new.” Solid lines indicate known interactions in the interaction space while dashed lines illustrate three kinds of unknown interactions (① unknown protein with known ligand, ② known protein with unknown ligand, ③ unknown protein with unknown ligand). (**b**) Features in bow-pharmacological space.

We hypothesised that chemogenomic modelling could profit from including not only information on the ligand and protein similarity but also explicitly on the pharmacological interaction space and hence the relationship between the ligands and the proteins (Fig. 1a). The combined information is composed of three sub-spaces, the shape of which resembles a bow tie, hence the name bow-pharmacological space. It covers a protein space that encodes protein sequence features, a ligand space that contains the fingerprints of chemical compounds, and an interaction space, coded by known interactions that connect the protein and ligand. Furthermore, we describe a novel prediction model by applying Bayesian Additive Regression Trees (BART) and other machine learning methods on these combined features from protein, ligand, and interaction information. Feature selection as well as subsampling experiments highlighted the utility of all the available descriptor subspaces and hence of the bow-pharmacological space (BOW space) newly developed here. Compared to other classical machine learning algorithms, the BART algorithm outperformed all tested methods and demonstrated good prediction power (94–99% accuracy on different datasets). Furthermore, BART can provide a quantitative description of the likelihood of predicted interactions and thereby provide an important measure of predictive uncertainty. In addition to retrospective analysis, we also highlight one exemplary prediction for a novel ligand of the KIF11 protein that was successfully validated using a docking simulation and subsequently confirmed by a crystallography study executed by an independent research group.

## Results

### Prediction based on bow-pharmacological space and BART

To predict the likelihood of protein-ligand interactions, information of both the known interactions and the non-interactions (positive and negative data) are required to build the training and testing datasets. For each protein-ligand pair (interaction or non-interaction), we coded 439 features in the bow-pharmacological space (Fig. 1). Based on these features, a statistical model was built to predict whether there was an interaction between a protein and a ligand. Due to the complexity of multiple possible interactions between proteins and ligands, we applied the Bayesian Additive Regression Trees (BART) to build the prediction model. BART is a Bayesian “sum-of-trees” model in which each tree is constrained by a regularized prior to be a weak learner, and fitting and inference are accomplished via an iterative Bayesian backfitting MCMC algorithm that generates samples from a posterior. BART enables full posterior inference including point and interval estimates of the unknown regression function as well as the marginal effects of potential predictors^14^ (see Methods).

To benchmark our approach against work by other researchers, we constructed our prediction models on published datasets by Yamanishi *et al*.^12^, Bleakley *et al*.^13^, Cao *et al*.^15^, Jacob *et al*.^16^ and He *et al*.^17^. When these datasets were combined, the numbers of enzymes, ion channels, GPCRs, and nuclear receptors were 664, 204, 95, and 26, respectively; the numbers of known drugs were 445, 210, 223, and 54, respectively; and the numbers of known interactions were 2926, 1476, 635, and 90, respectively.

**Figure 2:**
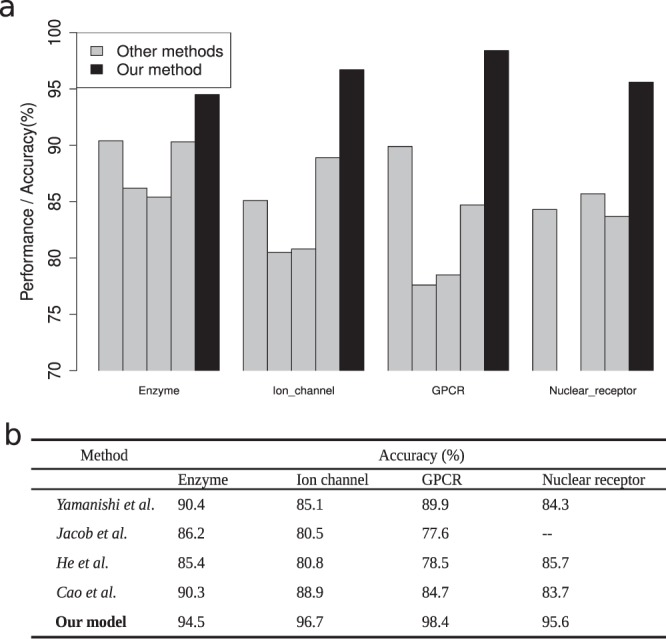
Comparison with other four prediction methods on the same dataset. (**a**) The prediction performance in enzymes, ion channels, GPCRs, and nuclear receptors were compared. Grey bars represent the performance (accuracy) of other methods, and black bars represent the performance of our method. (**b**) The performance values of our model and the other four methods.

The robustness of our model was assessed by a ten-fold cross-validation. We evaluated our model performances for sensitivity, specificity, accuracy, average receiver operating characteristic (ROC) curve, and the area under the curve (AUC) (see Methods). The accuracy of our model was 94.5%, 96.7%, 98.4%, and 95.6% for all four groups of proteins (enzymes, ion channels, GPCRs, and nuclear receptors). On the same dataset, our method performed better than other existing prediction methods that are based on chemical and genomic spaces^12^, protein sequence and drug topological structures^15^, a chemogenomics approach^16^, as well as functional group and biological features^17^ (Fig. 2).

**Figure 3:**
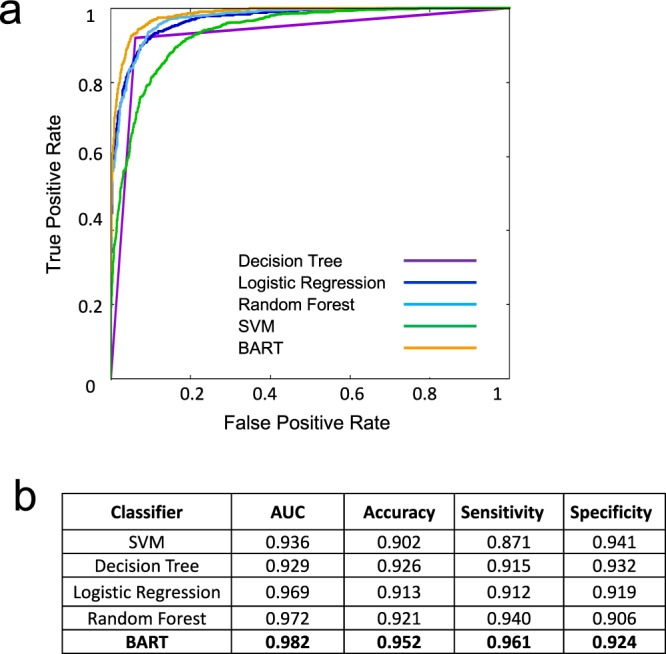
The prediction models with different machine learning methods on the entire dataset (Enzyme, Ion channel, GPCR, and Nuclear receptor). (**a**) The ROC curves of decision tree, logistic regression, random forest, SVM, and BART models. (**b**) The AUC, accuracy, sensitivity, and specificity of each model.

To directly compare the performance of BART with other established machine learning models, we used our training data (see Methods) to perform cross-validation experiments using random forest, support-vector machines (SVM), decision trees, and logistic regression. All models showed good performance (AUC > 0.9) when provided with the BOW space, while BART still showed superior performance (Fig. 3). Not surprisingly, the random forest–with arguably the most similar prediction architecture–showed the most similar performance, being outperformed by BART only in sensitivity. Simpler models such as decision trees showed lower performance on all applied measures. Interestingly, the well-established SVM showed the lowest accuracy, which was due to its low sensitivity but high specificity. Random forest, on the other hand, showed high sensitivity and low specificity. BART excelled in both measures and highlights the ability of the method to correctly classify both positive and negative data.

### Features in bow-pharmacological space

**Figure 4:**
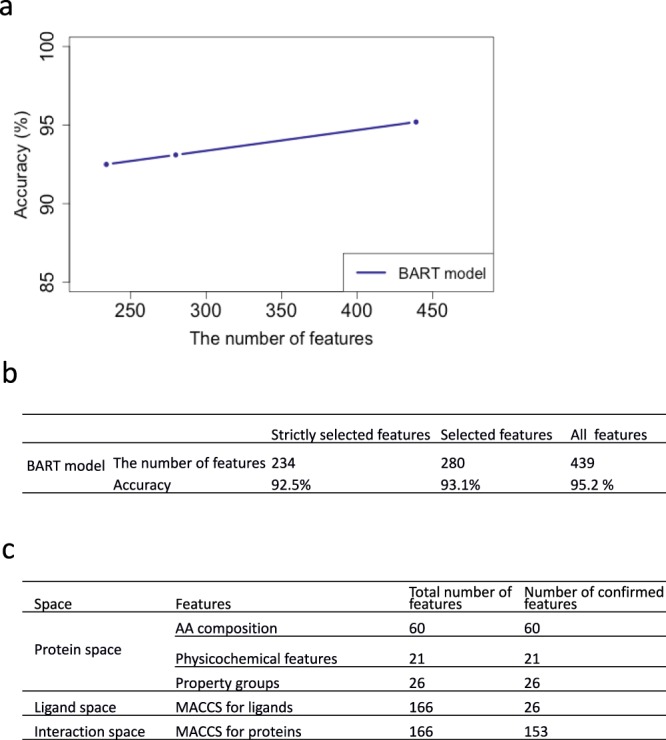
Prediction  models before and after feature selection on the entire dataset (Enzyme, Ion channel, GPCR and Nuclear receptor). (**a**) The prediction accuracy of models with different feature sizes. (**b**) The number of features and prediction performance. (**c**) The number of selected features in each part of bow-pharmacological space.

It is unknown whether all 439 features in our bow-pharmacological space contribute to the prediction and which features are more predictive than the others. Hence, we performed feature selection on the training data of the entire dataset (enzyme, ion channel, GPCR, and nuclear receptor) using Boruta, an algorithm that determines the relevance by using a wrapper approach built around a random forest classifier that compares real features to random probes^18^. Boruta divides features into three categories: “important”, “tentative”, and “unimportant.” First, we collected “important” features to form a feature dataset called “strictly selected features.” Next, we selected the “important” and “tentative” features to make up the “selected features.” The numbers of feature sets for individual models were summarized in Fig. 4. In general, “strictly selected features” contained almost half of all of the features, and “selected features” were close to two-thirds of all features. Importantly, we noted that every subspace (ligand, protein, and bow-interaction space) had conserved features, which highlights that the predictive accuracy depends on all descriptor subspaces. Moreover, this implies that all subspaces of the bow-pharmacological space contained relevant and non-redundant information (Fig. 4c). To test for the validity of the selected features through this approach, we tested the accuracy of all machine learning models here described when trained exclusively on the selected features, and saw only minor losses in performance. This highlights that the selected features are indeed able to decipher the interaction space using various different classification algorithms, and further increases the confidence in the novel descriptors proposed.

As a direct test of the utility of the bow-pharmacological interaction space, we decided to train all our machine learning models on all ligand and protein descriptors except the bow-interaction space. We observed a drop in all investigated performance measures, most notably a drop of around 10% of the AUC, highlighting the importance of the bow space to achieve the performance here reported. Interestingly, sensitivity seemed most affected, suggesting that the bow space is most useful to increasing the true positive rate.

We built prediction models with either “strictly selected features” or “selected features” for the three datasets in section 3 and compared the model performances. As shown in Fig. 4a,b, models with fewer features did not predict considerably better. Based on the increasing  number  in the feature sets (234, 280 and 439), the prediction accuracy was raised to 92.5%, 93.1% and 95.2%, respectively.

### Index-based physicochemical features (IPC) facilitates prediction and interpretation

Effective representation of proteins and ligands is essential for identifying drug-target interactions and it has previously been discussed that an optimal descriptor needs to be identified for a chemogenomic project^19^. In addition to our novel interaction space that extends the chemogenomic capabilities, we have also devised a new feature to represent proteins, called the index-based physicochemical feature (IPC). Previous protein representations fall into two general categories: structure-based and sequence-based. The structure-based representations rely on the knowledge of protein structure, which is not always available for most proteins; sequence-based representations only require information about the protein sequence, which is readily available. Typically, a sequence-based method uses the information of the amino acid composition of a protein, but neglects the sequence order of the amino acids in the polypeptide chain. To represent proteins with both the amino acid composition and sequence order information, we put forward IPC, a new feature that considers the effects from neighbouring amino acids. The effect of flanking amino acids to center amino acids declines as the distance of two amino acids increases along the protein sequence (see Methods).

**Figure 5:**
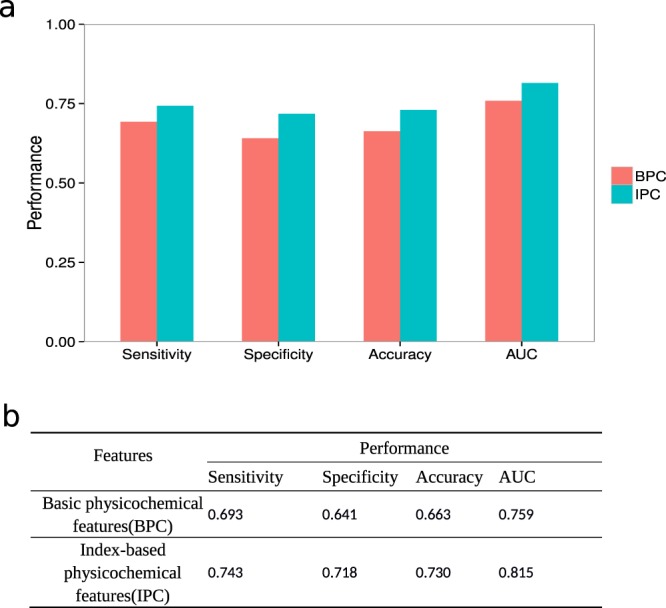
Comparison of the predictions based on basic physicochemical features (BPC) or index-based physicochemical features (IPC). (**a**) Sensitivity, specificity, accuracy, and AUC are plotted from left to right. Green bars represent the performance of prediction based on basic physicochemical features (BPC), red bars on index-based physicochemical features (IPC). (**b**) The performance values of BPC and IPC.

To evaluate the impact of IPC protein representation on model performance, we built one model with basic physicochemical features (BPC), which are classic sequence-based features for protein-related predictions and a second model with index-based physicochemical features (IPC). The model built with IPC features performed better than the model built with BPC in predicting protein-ligand interactions (Fig. 5). The IPC model achieved a prediction accuracy of 74.8% in comparison with the BPC model’s achieved 64.4%. This suggests that the proposed distance-aware IPC features were more informative than BPC for encoding protein sequence in protein-ligand prediction problems.

### Case studies

To test whether our prediction algorithm would identify any useful ligand-target interactions, we specifically investigated some of the most confident predictions. For example, based on our model, gamma-aminobutyric acid (CID000000119) was predicted with a high probability to interact with three proteins, but olfactory receptor 7G2 (ENSP00000303822) was put forward as the most likely interaction candidate protein (a segment of results generated by our model is tabulated in Supplement Table S1). The interaction between CID000000119 and 7G2 was revealed in the literature^20^ but had not been collected in the database yet.

**Figure 6:**
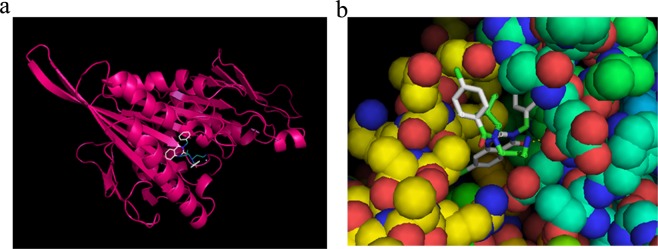
Docking simulation for G7X and kinesin-like protein KIF11. (**a**) Kinesin-like protein KIF11 is in pink and G7X in white and green. G7X is likely to bind to the protein pocket. (**b**) Zoomed-in close-up of the binding zone. Spheres represent proteins, and stick represents G7X.

To illustrate that our model is able to search for new ligands of important target proteins, we present a case study to predict new interacting ligands for kinesin-like protein, KIF11. KIF11 is a cytoskeletal protein that belongs to the kinesin-like protein family and plays a role in chromosome positioning, centrosome separation, and bipolar spindle establishment during cell mitosis. KIF11 is inhibited by certain small molecules such as Monastrol, a prototype anti-cancer drug that selectively inhibits a mitotic kinesin Eg5, several derivatives of which are currently under clinical trials and being investigated in the field of malignant tumour study^21^. Based on our model’s prediction, KIF11 interacts with ispinesib mesilate (G7X) with a probability of 0.92. To verify the prediction, we performed a docking simulation and literature search. In the docking result, G7X was obviously bound to KIF11 (Fig. 6). The binding affinity calculated by AutoDock Vina (1.1.2) is −9.5 kcal/mol, which falls within the conventional binding energy interval of −9 to −12 kcal/mol. The prediction and simulation results were further validated recently by an independent group using a crystallography method. Their results showed the same pose as the docked pose^22^, attesting to the predictive accuracy of our model.

## Discussion

Protein-ligand interactions are fundamental for myriad processes occurring in living organisms. Our investigation into these interactions is therefore promising for our understanding of the biochemical underpinning of cellular systems and of perturbations into these systems, and constitutes a major step in drug discovery research. With the development of sophisticated computer algorithms, protein-ligand interactions have been increasingly deconvoluted by *in silico* approaches. Our study described the development of a machine learning approach based on a new class of descriptors as well as a novel algorithm to accurately predict protein-ligand (drug-target) interactions.

For the first time, we applied the Bayesian additive regression trees (BART) algorithm on a uniform space that encodes feature information from proteins and ligands, a classical chemogenomic approach, but here for the first time also include an interaction space that encodes for known protein-ligand interactions. This space was constructed by relying on average fingerprints—a concept that has been underexplored in the computational drug design community and has most notably been applied as median molecules in *de novo* design^23–25^ as well as implicitly when using clustering approaches^26,27^. This information space was coined the bow-pharmacological space. It encapsulates essentially non-redundant and relevant information for predicting interaction between proteins and potential ligands or vice versa and we showed a significant increase in performance over various established machine learning algorithms when supplied with the novel descriptor, highlighting its utility (Fig. 1). Furthermore, we also developed novel protein target descriptors that included predicted tertiary structure and showed an improved performance over two-dimensional protein descriptors. We foresee an increased interest in using such types of descriptors by other researchers in pharmaceutical and chemical biology research.

In our model, BART, a non-parametric Bayesian regression approach, is applied. It provides a reliable posterior mean and interval estimates of the true regression function as well as the marginal effects of potential predictors^14^, while many other binary classification tools (e.g., KNN, SVM)^28,29^ simply produce a binary yes-or-no result. For the interaction within a protein-ligand pair, BART generates a probabilistic scoring of the likelihood of the interaction. An *in silico* probabilistic evaluation of the likelihood of interactions could serve as an initial filtering step to select the most probable candidates out of a pool of hundreds or even thousands, thus lowering the experimental cost and time.

Our approach extends our knowledge of potential ligands for a specific protein, and proteins that interact with a specific ligand are useful in drug discovery efforts to identify yet undiscovered protein-ligand interactions. In addition, the probability index of protein-ligand pairs can be used for filtering and stratifying multiple drug candidates, as well as for evaluating the off-target effects of specific drugs and other protein-ligand interactions. With the high predictive accuracy and high-throughput performance of our prediction algorithm, we envision that more drugs will be able to be evaluated and developed more rapidly, and a deeper understanding of drug effects and drug targets will be achieved.

## Models and Methods

### Construction of bow-pharmacological space

All features in the bow-pharmacological space are summarized in Fig. 1b. In the protein space, we considered three main feature types for comprehensively representing a protein. These feature types include the amino acid composition, physicochemical features of the protein, and property groups in the polypeptide sequence. Further, these types were subdivided into ten feature sets designated F1, F2, …, F10. The composition vector (CV, as F1) contains information about the amino acid composition of the primary protein sequence, but not its relative position. To describe both the composition and the relative position of amino acids in the protein sequence, we used the composition moment vector (CMV, as F2).

In addition, we included three different types of physicochemical features: first, basic physicochemical features (BPC, as F3) such as hydrophobicity, charge, and polarity serve as a classic description of protein sequence, which has performed well for many protein-related prediction problems^30–32^; second, the neighbourhood-based physicochemical feature (NPC, as F4) complemented the BPC by combining the target amino acid site and its two neighbours; and third and most importantly, the index-based physicochemical feature (IPC, as F5) was constructed with the assumptions that each site on the protein sequence had an effect on others and that the effects were related to the protein composition (see Methods). Regarding amino acid positions, amino acids that were close to the primary sequence could be part of flexible loops and therefore not close in 3D space. Conversely, amino acids that were far apart might form a pocket. IPC is the descriptor that incorporated the secondary/tertiary structure (prediction). Additionally, we incorporated five feature sets for the protein property groups, including the R groups (RG, as F6), the electronic groups (EleG, as F7), the exchange groups (ExG, as F8), the hydrophobic groups (HG, as F9) and the side chain groups (SCG, as F10).

In the ligand space, we adopted the MACCS fingerprint, one of the most widely used “structural fingerprints” based on pre-defined chemical substructures^33^. MACCS has 166-bit structural key descriptors, each of which is associated with a *SMARTS* pattern that represents a functional group or test of a combination of substructure^34^.

**Figure 7:**
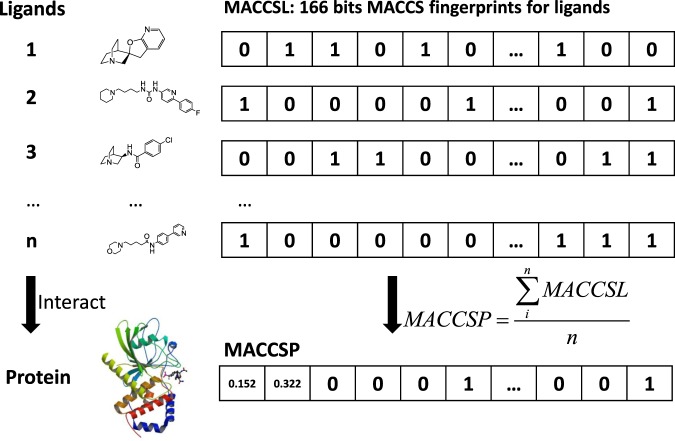
The scheme of generating MACCSP. Ligands, MACCS keys, and the function for generating MACCSP are illustrated. Note that the numbers in MACCSL and MACCSP are artificial, not real numbers.

In the protein-ligand interaction space, the links that represented the known interactions between proteins and their ligands were quantified. As shown in Fig. 7, known ligands of each protein were coded by the MACCS keys; these keys were averaged to generate a unique fingerprint that represented the known links between each protein and the ligands. We named this feature MACCSP.

### Gold standard dataset

The interactions between ligands and target proteins were retrieved from the KEGG BRITE^35^ and DrugBank databases^36^. The number of known interactions are 5,125 in total; 2926, 1476, 635, and 90 for enzymes, ion channels, GPCRs and nuclear receptors, respectively. The number of known proteins/drug targets in each category was 664, 204, 95, and 26, respectively. Chemical structures of the drugs and ligands were obtained from the DRUG and COMPOUND Sections in the KEGG LIGAND database^35^. Amino acid sequences of the target proteins were obtained from the NCBI database. Taken together, 5,125 interactions were treated as the positive dataset.

The negative dataset (non-interactions) was composed of the proteins and ligands which were not in the 5,125 interactions. The protein pool was generated by eliminating 1,051 proteins in the positive dataset from the 16,267 human-origin proteins in Swiss-Prot (2012). The ligand pool was generated by eliminating the ligands in the positive dataset from the 525,766,279 ligands in the STITCH database (2012). After mixing the positive and negative datasets, a randomly selected 70% of the data was used for training and the other 30% was used for testing. In our study, both 10-fold cross-validation and independent testing were used to assess model performance.

### Coding features in bow-pharmacological space

#### Protein space

**Feature 1**: Composition vector (CV, 20 dimensions).

*CV*_*i*_ denotes the percentage composition of amino acid (AA) *i* in the protein sequence:

*CV*_*i*_ = (number of amino acid *i* in the sequence)/(total number of AA’s in the sequence).

20 amino acids were coded in alphabetical order: A, C, D, E, F, G, H, I, K, L, M, N, P, Q, R, S, T, V, W, Y, and were denoted AA_1_, AA_2_, …, AA_20_, respectively.

**Feature 2**: First and second order composition moment vector (CMV, 40 dimensions).

The composition moment vector of a protein was defined as follows:

For *k* = 1, ${x}_{i}^{(1)}$ is the *i-*th entry of the first-order composition moment vector,

$$CM{V}_{i}^{1}={x}_{i}^{(1)}=\frac{1}{N(N-1)\,\ldots \,(N-k)}\sum _{j}\,{({n}_{ij})}^{1}$$

and for *k* = 2, ${x}_{i}^{(1)}$ is the *i-*th entry of the second-order composition moment vector,

$$CM{V}_{i}^{2}={x}_{i}^{(2)}=\frac{1}{N(N-1)\,\ldots \,(N-k)}\sum _{j}\,{({n}_{ij})}^{2}$$

where *CMV* is the composition of the *i-*th AA in the sequence, *N* is the length of the AA sequence, *n*_*ij*_ is the *j-*th position of AA_*i*_ and *k* is the order of the composition moment vector.

The first and second orders of CMV were used, while the zeroth order reduces to the composition vector (CV, feature 1).

**Feature 3**: Basic physicochemical features (BPC, 7 dimensions).

In this study, seven physicochemical properties were chosen from AA index. They include hydrophobicity, charge, polarity, volume, flexibility, isoelectric point, and refractivity. For each of these properties, the basic physicochemical feature is calculated by $BPC=\sum _{i=1}^{N}{P}_{i}$, where *P*_*i*_ is the relevant physicochemical property of the *i-*th amino acid in the sequence.

**Feature 4**: Neighbourhood-based physicochemical features (NPC, 7 dimensions).

The seven physicochemical properties in the NPC are the same as those in the BPC. For each property, the NPC feature considers the effect of the properties of its neighbouring AA and is calculated by $NPC=\sum _{i=1}^{N}|{({P}_{i})}^{2}-{P}_{i-1}\times {P}_{i+1}|$, where *N* is the length of the protein sequence, and *P*_*i*_ is the concerned physicochemical property of the *i-*th amino acid in the sequence.

**Feature 5**: Index based physicochemical features (IPC, 7 dimensions).

The seven physicochemical properties used in IPC are the same as those in the BPC and the NPC. For each property, the IPC feature is calculated in three steps.

Step 1:

$${P}_{1}({R}_{i})=\frac{{P}_{1}^{0}({R}_{i})-{P}_{1}^{0}}{SD({P}_{1}^{0})}$$

where *P*_1_(*R*_*i*_) is the original value of physicochemical feature 1 (seven in total, first is hydrophobicity). ${P}_{1}^{0}$ is the average of the basic physicochemical feature 1 over the 20 AAs, and $SD({P}_{1}^{0})$ is the corresponding standard deviation. *P*_*i*_ is also calculated for *i* in 2, …, 7 (six other physicochemical features: charge, polarity, volume, flexibility, isoelectric point and refractivity).

Step 2:

$${J}_{i,i+k}={[{P}_{1}({R}_{i+1})-{P}_{1}({R}_{i})]}^{2}$$

$${\delta }_{k}=\frac{1}{N-k}\sum _{i=1}^{N-k}{J}_{i,i+k}$$

where *k* is the interval between two amino acids, *k* ∈ [1, *N* − 1]; N is the number of amino acids in the sequence. *δ*_*k*_ is the *k*-th correlation factor that reflects the sequence order correlation between all the *k*-th most contiguous residues.

For example, with *k* = 1, we have

$${\delta }_{1}=\frac{1}{N-1}({J}_{1,2}+{J}_{2,3}+{J}_{3,4}+\ldots +{J}_{N-1,N})$$

and with *k* = 2, we have

$${\delta }_{2}=\frac{1}{N-2}({J}_{1,3}+{J}_{2,4}+{J}_{3,5}+\ldots +{J}_{N-2,N})$$

Accordingly, all the *J* and *δ* values can be calculated.

Step 3:

After calculating all the *J* and *δ*_*k*_, calculate the IPC,

$$IPC=\frac{{\sum }_{k=1}^{N-1}{\delta }_{k}}{N-1}$$

**Feature 6**: R group features (RG, 5 dimensions).

There are five types of protein R groups. *RG*_*i*_ is the percentage of all amino acids in the sequence that have *R* groups of type *i*, where *i* = 1, 2, …, 5. The case of *i* = 1 corresponds to non-polar aliphatic AAs (A, G, I, L, M, V), *i* = 2 to polar uncharged AAs (C, N, P, Q, S, T), *i* = 3 to positively charged AAs (H, K, R), *i* = 4 to negative AAs (D, E), and *i* = 5 to aromatic AAs (F, W, Y).

**Feature 7**: Electronic group features (EleG, 5 dimensions).

*EleG*_*i*_ is the percentage composition of electronic group *i* in the sequence, where *i* = 1, 2, …, 5. The case in which *i* = 1 corresponds to electron donor AAs (A, D, E, P), *i* = 2 to weak electron donor AAs (I, L, V), *i* = 3 to electron acceptor AAs (K, N, R), *i* = 4 to weak electron acceptor AAs (F, M, Q, T, Y), and *i* = 5 to neutral AAs (G, H, S, W).

**Feature 8**: Exchange group features (ExG, 6 dimensions).

Exchange groups were clustered by the conservative replacements of amino acids during evolution. *ExG*_*1*_ corresponds to the amino acid C; *ExG*_*2*_ to A, G, P, S, T; *ExG*_*3*_ to D, E, N, Q; *ExG*_*4*_ to H, K, R; *ExG*_*5*_ to I, L, M, V; and *ExG*_*6*_ to F, W, Y.

**Feature 9**: Hydrophobicity group features (HG, 4 dimensions).

Hydrophobicity groups were formed according to the water-soluble side chains of amino acids. *HG*_*i*_ is the percentage composition of hydrophobicity group *i* in the sequence. The case *i* = 1 corresponds to hydrophobic AAs (A, C, F, G, I, L, M, P, V, W, Y), *i* = 2 to hydrophobic basic AAs (H, K, R), *i* = 3 to hydrophobic acidic AAs (D, E), and *i* = 4 to hydrophobic polar with uncharged side chain AAs (N, Q, S, T).

**Feature 10**: Side chain group features (SCG, 6 dimensions).

Side chain groups were based on the attributes of side chains including molecular weight, polarity, aromaticity, and charge. *SCG*_*i*_ is the percentage composition of side chain group *i* in the sequence. The case in which *i* = 1 corresponds to tiny side chain AAs (A, G), *i* = 2 to bulky side chain AAs (F, H, R, W, Y), *i* = 3 to polar-uncharged AAs (D, E), *i* = 4 to charged side chain AAs (D, E, H, I, K, L, R, V), *i* = 5 to polar side chain AAs (D, E, K, N, Q, R, S, T, W, Y), and *i* = 6 to aromatic side chain AAs (F, H, W, Y). Although this feature and Feature 6 were both based on the R groups of amino acids, they are different in division criteria and biological meaning.

#### Ligand space

**Feature 11**: MACCS for ligands (MACCSL, 166 dimensions).

Each ligand was represented with a MACCS key fingerprint, which was calculated with molecular operating environment (MOE). MACCS encoded the molecular structure in 166 bits (binary digits). Each bit in a structural fingerprint corresponds to the presence (1) or absence (0) of a specific substructure in the molecule.

#### Protein-ligand interaction space

**Feature 12**: MACCS for proteins (MACCSP, 166 dimensions).

To encode the information of known protein-ligand interactions, we first collected all the known ligands for each specific protein and then added up the MACCSL values of these interacted ligands. Finally, the sum was divided by the total number of connected ligands.

### Feature selection by Boruta

The Boruta algorithm is a wrapper method built around the random forest classification algorithm^18^. Random forest is a category of ensemble methods in which classification is performed by voting of multiple unbiased weak classifiers (decision trees). These decision trees are independently developed on different samples drawn independently and randomly from the training set. A random permutation of each feature was performed, and the resultant loss of accuracy of the classification was measured for each tree to infer the importance of the feature.

### BART and other machine learning models

Bayesian Additive Regression Trees (BART) is a Bayesian tree ensemble method for non-parametric learning. The unique characteristic of BART is a regularization prior that encourages the decision trees in the Bayesian tree ensemble to be small in size. The sum of the resultant trees, each of which is a weak learner, combines to be a non-parametric model that explains and predicts the relation between the predictors and responses. The trees and the corresponding weights are developed with the boosting algorithm implemented through Markov chain Monte Carlo (MCMC).

We used the R package Bart to implement this method. BART was defined by a statistical model: a prior and a likelihood. The features proposed above are used as input into the BART algorithm. Essentially, BART first constructed a simple weak learner by a prior and then built a Bayesian “sum-of-trees” model. To fit the model, BART employed a tailored version of Bayesian backfitting Markov chain Monte Carlo (MCMC) method that interactively constructed and fitted successive residuals^37^. The probability values above 0.5 generated by BART were classified to “interaction” group, and the values equal/below 0.5 were classified to “non-interaction” group.

Besides BART, other machine learning methods were applied as well, including logistic regression^38^, support vector machine (SVM)^39^, decision tree, and random forest. Logistic regression is a statistical method for analyzing a dataset in which there are one or more independent variables that determine an outcome, and is used for estimating the probability of an event^38^. Support vector machine (SVM) efficiently performs a non-linear classification using what is called the kernel trick, implicitly mapping inputs into high-dimensional feature spaces to build a maximum margin hyperplane. A decision tree is a decision support tool that uses a tree-like graph or model of decisions and their possible consequences. Random forest is a meta-estimator that fits a number of decision tree classifiers. Each tree gives a classification, and we say the tree “votes” for that class. The forest selects the classification having the most votes in the forest. We used Python along with a machine learning package, scikit-learn (specifically linear_model.LogisticRegression with parameter C = 1e5) to implement logistic regression. The SVM models were built based on the libsvm package from Sklearn (svm.SVC), where gamma was set at 0.0001 and C was set at 100. We implemented decision trees with the function tree. DecisionTreeClassifier from the sklearn package. We used the RandomForestClassifier class in Sklearn.ensemble along with the number of jobs equal to six for algorithm implementation.

### Performance measurements

We conducted a 10-fold cross-validation and independent testing to evaluate the predictive performance of the models. A confusion matrix was applied to calculate sensitivity, specificity, and overall accuracy of our classifiers. Accuracy = (TP + TN)/(TP + FP + TN + FN), Sensitivity = TP/(TP + FN), and Specificity = TN/(TN + FP), where TP is the number of true positives, TN is true negatives, FP is false positives, and FN is false negatives. Furthermore, Receiver Operator Characteristic (ROC) curves were plotted to depict relative trade-offs between accuracy and coverage with TP on the *y*-axis and FP on the *x*-axis^40^. The area under the ROC curve (AUC) was also calculated as a measurement of performance^41^.

## Supplementary information

S1 Table
