# Supplementary material for: Predicting protein-ligand interactions based on bow-pharmacological space and Bayesian additive regression trees
Source: Sci Rep. 2019 May 22;9:7703. doi: 10.1038/s41598-019-43125-6 (PMC6531441; doi:10.1038/s41598-019-43125-6)
Supplement: Supplementary file 1 — S1 Table [file 41598_2019_43125_MOESM1_ESM.pdf]

Predicting protein-ligand interactions based on bow-pharmacological space and Bayesian additive regression trees

Li Li[1,2,3], Ching Chiek Koh[4], Daniel Reker[5], J.B. Brown[6], Haishuai Wang[7,8], Nicholas Keone Lee[4], Hien-haw Liow[9], Hao Dai[1,10], Huai-Meng Fan[1], Luonan Chen[10,11], and Dong-Qing Wei[1§]

[1] College of Life Science and Biotechnology, Shanghai Jiao Tong University, 800 Dongchuan Road, Shanghai 200240, China

[2] Cellular Networks and Systems Biology, University of Cologne, CECAD, Joseph-Stelzmann-Strasse 26, Cologne 50931, Germany

[3] Department of Genetics, Harvard Medical School, Boston, MA 02115, USA

[4] Wellcome Sanger Institute, Wellcome Trust Genome Campus, Hinxton, Cambridge CB10 1SA, UK

[5] Koch Institute for Integrative Cancer Research, Massachusetts Institute of Technology, Cambridge, MA 02139 (USA)

[6] Laboratory of Molecular Biosciences, Life Science Informatics Research Unit, Kyoto University Graduate School of Medicine, Kyoto 606-8501 Japan

[7] Department of Computer Science and Engineering, Fairfield University, Fairfield, Connecticut 06824, USA

[8] Department of Biomedical Informatics, Harvard Medical School, Boston, MA 02115, USA

[9] Center for Genome Sciences and Systems Biology, Washington University, St. Louis, MO 63130, USA

[10] Key Laboratory of Systems Biology, Innovation Center for Cell Signaling Network, Institute of Biochemistry and Cell Biology, Shanghai Institutes for Biological Sciences, Chinese Academy of Sciences, Shanghai 200031, China

[11] School of Life Science and Technology, ShanghaiTech University, Shanghai 201210, China

[§]Corresponding author

Supplement Table S1 A segment of the result generated by our model.

**Ligands**	**Proteins**	**Probability**
CID000000051	ENSP00000252799	0.9813413
CID000000051	ENSP00000396814	0.9962303
CID000000119	ENSP00000282369	0.9135653
CID000000119	ENSP00000303822	0.9984301
CID000000119	ENSP00000331912	0.9137092
**…**	…	…
